# Can Patients Self-Identify Gait Disturbances After Lower Extremity Trauma? Enhancing Patient Engagement in Their Care

**DOI:** 10.3390/jcm15031175

**Published:** 2026-02-03

**Authors:** Tyler Forbes, Joseph Cavataio, Andrew Robinson, Abdel Kareem Hilo, Alyxandra Camello, Anagha Purushotham, Rahul Vaidya

**Affiliations:** 1 School of Medicine, Wayne State University, Detroit, MI 48201, USA; andrew.robinson4@med.wayne.edu (A.R.); hy9254@wayne.edu (A.K.H.); rahvaidya2012@gmail.com (R.V.); 2 Department of Orthopedic Surgery, Detroit Medical Center, Detroit, MI 48201, USA; jcavataio@tulane.edu (J.C.); bellecamello@gmail.com (A.C.); anaghapurushotham1@gmail.com (A.P.)

**Keywords:** patient engagement, PROMIS, gait disturbance, rehabilitation, lower extremity trauma

## Abstract

**Background and Objectives:** Orthopedic patients recovering from lower extremity trauma frequently experience gait disturbances that affect mobility, independence, and quality of life. Engaging patients in recognizing their own gait abnormalities may enhance participation in rehabilitation and improve functional outcomes. The purpose of this study to assess if patients recovering from lower extremity trauma can self-recognize the presence or absence of a gait abnormality, and if the PROMIS physical function scores correlate with gait abnormality. **Materials and Methods:** An IRB-approved prospective cross-sectional study was conducted at a Level One U.S. trauma center, assessing gait disturbance in patients recovering from lower limb surgery. Participants completed the PROMIS physical function survey along with self-assessing the presence or absence of a limp, then received a clinical gait assessment by a clinician. Of 243 patients screened, only those with an isolated lower limb injury, status post-operative trauma care, and able to ambulate unassisted were included. This yielded a final study cohort of 66 patients. **Results:** Post-lower extremity trauma patients were able to self-identify the presence (95.92% *p* < 0.00001) or absence 89.47% (*p* < 0.00001) of a gait abnormality. There was a statistically significant difference in PROMIS physical function t-scores between patients with and without a limp, 37.2 and 44.4 (*p*-value 0.00012), respectively. **Conclusions:** Patients recovering from lower extremity trauma can effectively identify the presence or absence of gait disturbances. Limp recognition was associated with lower PROMIS Physical Function scores. Promoting patient awareness and involvement in tracking their recovery may enhance engagement, guide clinical decision-making, and support better rehabilitation outcomes.

## 1. Introduction

Gait disturbances are a common and often persistent complication after lower extremity trauma, impacting mobility, independence, and quality of life. A normal gait works in coordinated, energy-efficient movements; in contrast, an abnormal gait such as a limp places greater physical demand on the body, may carry social stigma, and is associated with ongoing pain and functional limitation [1,2,3,4]. Orthopedic trauma surgery from lower extremity injuries aims to restore normal, painless ambulation, but successful rehabilitation depends on timely recognition and correction of gait abnormalities.

Traditionally, gait assessment is conducted by trained clinicians during in-person visits. However, the growing focus on patient-centered care underscores the importance of engaging patients as active participants in their own recovery. Early recognition of functional changes by patients themselves may facilitate faster intervention, improve adherence to rehabilitation, and support better long-term outcomes [5,6,7,8,9]. The Patient-Reported Outcomes Measurement Information System Physical Function (PROMIS PF) survey is a validated tool widely used to quantify musculoskeletal function, but its relationship to patient self-perception of gait disturbances after trauma has not been clearly defined [10,11].

The current literature is limited in identifying the association between patients’ self-recognition of a limp and clinician-observed gait status, nor has it examined how this self-awareness correlates with validated functional outcome measures. Prior clinical research has demonstrated that gait disturbances can be reliably identified through visual observation alone; for example, Robinson et al. (2023) showed that both medical professionals and laypersons could accurately recognize gait disturbances from video observation [12].

In addition, prospective longitudinal clinical research using instrumented insole gait monitoring after tibial and malleolar fractures found that objective gait parameters often improve earlier than patients’ perceived functional recovery, suggesting that patients’ subjective assessment may not always align with objective measures, but could nevertheless provide meaningful information about rehabilitation progress [13]. Understanding whether patients can accurately assess their own gait has important implications for postoperative care models, particularly in telemedicine and resource-limited settings. If patient self-assessment proves reliable, it could be integrated into remote monitoring strategies to streamline follow-up and facilitate earlier rehabilitation adjustment.

The primary aim of the study was to analyze whether patients can accurately identify their own gait abnormalities following post-operative lower extremity trauma surgery. PROMIS-PF scores were also analyzed to look for correlation with limp status. The hypothesis of this study is that patients can identify their own limp with high accuracy, and limp presence is associated with lower functional scores.

## 2. Materials and Methods

This study was approved by the institutional review board as a prospective cross-sectional study conducted at a Level I U.S. trauma center. This study was designed as a prospective cross-sectional analysis to evaluate patient self-perception of gait disturbance at a single postoperative time point during routine outpatient follow-up. A cross-sectional design was selected to reflect real-world clinical encounters, where gait assessment and functional status are commonly evaluated during scheduled visits rather than longitudinal gait laboratory assessments.

The study enrolled 243 patients who were status post lower extremity trauma surgery. Patients were excluded if they had experienced polytrauma, were unable to ambulate without mechanical assistance (braces, crutches, canes, walker, and scooter), were unable to complete the survey, or had concurrent abdominal, thoracic, or upper extremity trauma. Patients with prior gait assessment by a clinician were also excluded. A total of 66 of the 243 enrolled patients met inclusion criteria. Verbal informed consent was obtained from each patient prior to survey administration.

Demographic information, type of surgery (ICD-10 and CPT codes), time from surgery, and health status were obtained for each patient (Table S1). The survey included the PROMIS Physical Function short form questionnaire and a customized section regarding ambulation and the presence of a limp (yes/no). Patient self-assessment of gait was obtained using the standardized question, “Do you think you have a limp?” with binary yes/no response options. Patients completed the self-reported survey prior to undergoing clinical gait assessment. Following survey completion, each patient’s gait was independently assessed by a single, fellowship-trained orthopedic surgeon with nearly 40 years of clinical experience who was blinded to patient self-reported responses. Patients were observed ambulating unassisted in a clinic hallway at a self-selected walking speed over a standardized distance of 60 feet, consisting of 30 feet in one direction followed by a return over the same distance. Gait assessment was completed with shoes on. If patients were unable to complete the full distance due to discomfort or fatigue, gait assessment was performed over a shorter distance sufficient to allow clinical evaluation.

Clinical gait assessments performed utilized a standardized protocol that focused on gait symmetry, cadence, and stance and swing phases, with attention to observable deviations from normal walking mechanics. For the purposes of this study, a limp was defined as any observable deviation from normal, symmetric gait, including antalgic gait, asymmetric stance time, or compensatory trunk or limb movements. All clinician-determined gait findings were then compared with patient self-reported survey responses.

Clinical gait assessment was performed by a board-certified attending orthopedic trauma surgeon during routine outpatient evaluation. Patients were observed ambulating unassisted in a clinic hallway at a self-selected walking speed over a standardized distance of 60 feet, consisting of 30 feet in one direction followed by a return over the same distance. The gait analysis was completed with shoes on. If patients were unable to complete the full distance due to discomfort or fatigue, gait assessment was performed over a shorter distance sufficient to allow clinical evaluation. Assessment focused on gait symmetry, cadence, and stance and swing phases, with attention to observable compensatory movements. For the purposes of this study, a limp was defined as any observable deviation from normal, symmetric walking mechanics, including antalgic gait, asymmetric stance time, or compensatory trunk or limb movements.

Gait was recorded dichotomously as either “limp present” or “no limp present,” reflecting real-world clinical decision-making rather than formal gait laboratory analysis. Based on comparison with clinician gait assessment, responses were classified as true positive (patient and clinician agree on the presence of a limp), true negative (agreement on absence of a limp), false positive (patient reports a limp but clinician observes none), or false negative (patient reports no limp but clinician observes a limp).

The PROMIS Physical Function short form was fully completed by all participants. The instrument consists of eight items, yielding raw scores that range from 8 (lowest function) to 40 (highest function) [11]. These raw scores are then converted into standardized T-scores utilizing the, with a mean of 50. A PROMIS PF T-score of 60.1 represents optimal physical function, indicating the individual can carry out daily activities without difficulty.

The reference standard was the clinician-observed limp status, which was used to create a 2 × 2 contingency table and calculate sensitivity, specificity, positive predictive value (PPV), negative predictive value (NPV), and overall accuracy, each with 95% confidence intervals (Wilson method). Agreement beyond chance was quantified using Cohen’s kappa with 95% confidence intervals. To address heterogeneity in follow-up timing, the same diagnostic accuracy metrics and kappa were calculated separately for patients following up ≤52 weeks and >52 weeks post-surgery, based on evidence demonstrating gait quality can continue to improve up to 12 months post-surgery [14,15].

A secondary analysis was conducted to calculate a PROMIS PF T-score cutoff predictive of limp with at least 85% specificity. A receiver operating characteristic (ROC) curve was generated for the T-scores with clinician-observed limp status as the binary outcome. The area under the curve (AUC) was calculated with 95% confidence intervals. Diagnostic performance at the calculated cutoff was summarized using the metrics reported in the primary analysis. All statistical analyses were performed using R version 4.3.1 (R Foundation for Statistical Computing, Vienna, Austria) and RStudio Version 4.3.1 (Posit team, 2025).

## 3. Results

The mean (±SD) age of patients in this study was 49.0 (±15.6) years, with 55% being male. The median (Q1–Q3) time from surgery patients were assessed was 36.0 (12.0–85.8) weeks. The distribution of injury types primarily consisted of fractures of the tibia (27.3%), fractures of the ankle (18.2%), fractures of the femur (10.6%), knee extensor mechanism injury (7.6%), tibial pilon fracture (7.6%), and the remainder of patients fell into sparse categories, as shown in Table S1.

There were 46 patients in the true positive category, where the patient and clinician agreed on the presence of a limp (Table 1). Four patients were in the false positive category, incorrectly self-identifying as having a limp. There were 16 patients in the true negative category and one patient answered in the false negative category.

Median (Q1–Q3) time after surgery in patients with a true limp was 36 (12–57) weeks (maximum of 140 weeks) was similar to patients without a limp at 47 (15–92.5) weeks (maximum of 495 weeks) (*p* = 0.30). The three patients in the false positive category were surveyed more than one year after surgery. The one patient in the false negative category was surveyed at 10 weeks from surgery.

### 3.1. Patient and Clinician Agreement Metrics for Identification of Limp

Patients accurately self-diagnosed the presence or absence of a limp at a high rate (93.9%, 95 CI: 85.4–97.6). Patients demonstrated excellent sensitivity in self-identifying a limp (97.9%, 95 CI: 88.9–99.6%) and moderate/high specificity in correctly identifying the absence of a limp (84.2%, 95 CI: 62.4–94.5%). The NPV in self-identifying a limp was excellent (94.1%) as was the PPV (93.9%) (Table 2). Agreement between patient and clinician was almost perfect (Cohen’s κ: 0.85, 95 CI: 0.70–0.99).

There were 38 patients evaluated less than one year from surgery and 28 evaluated more than more after surgery. Overall accuracy was high in both groups, at 97.4% (95 CI: 86.5–99.5%) in surveys <1 year post-surgery and 89.3% (95 CI: 72.8–96.3%) in surveys >1 year post-surgery. Patients in both groups demonstrated excellent sensitivity; however, specificity differed greatly at 100% (95 CI: 77.2–100%) and 50.0% (95 CI: 18.8–81.2%) in surveys <1 year and >1 year post-surgery, respectively. The PPV remained robust in surveys >1 year post-surgery (88.0%, 95 CI: 70.0–95.8%). Agreement between patient and clinician was almost perfect in patients surveyed <1 year post-surgery (Cohen’s κ: 0.94, 95 CI: 0.83–1.00) and was moderate in those surveyed >1 year post-surgery (Cohen’s κ: 0.61, 95 CI: 0.23–0.99).

### 3.2. PROMIS PF T-Score and Limp Status

Patients without a limp had a significantly higher mean PROMIS PF T-Score at 44.5 (±SD: 8.8) compared to patients with a limp 37.2 (±SD: 5.5) (*p* = 0.002). The optimal t-score cutoff was calculated to be 36.7, resulting in an accuracy of 63.6% (95 CI: 51.6–74.2%), sensitivity of 53.2% (95 CI: 39.2–66.7%), and a specificity of 89.5% (95 CI: 68.6–97.1%). This cutoff resulted in a high PPV (92.6%, 95 CI: 76.6–97.9%) and low/moderate NPV (43.6%, 95 CI: 29.3–59.0%).

## 4. Discussion

This study evaluated whether patients recovering from lower extremity trauma can self-identify gait disturbances. Only patients who had surgery, were weight-bearing, and did not use a walking aid were included in this study. Evidence has suggested both clinicians and laypeople can easily identify gait disturbances. Accordingly, this study utilized a binary definition of limp to evaluate and simplify the feasibility of patient self-recognition at a clinically meaningful threshold during routine recovery. If patients can accurately recognize their own limp, they may take a more active role in their care and improve health outcomes through self-management [16].

The present study intentionally focused on a narrowly defined postoperative cohort to examine patient self-recognition of gait disturbance in the absence of obvious external cues or prior training. Patients requiring assistive devices were excluded to avoid circular classification, as device use inherently implies gait abnormality. Exclusion of polytraumatized patients minimized confounding from non-lower extremity injuries that could independently influence gait mechanics or functional self-perception. Additionally, patients with prior formal gait assessment experience were excluded to evaluate self-awareness of gait disturbance in individuals without specialized training, reflecting the experience of the general population. This approach strengthened internal validity for the study’s primary objective.

Patients in this study cohort recovering from lower extremity trauma surgery could accurately self-identify a limp with high sensitivity (97.9%) and specificity (84.2%). The PPV and NPV values reflect accuracy levels useful to a clinician assessment of patient ambulation. An accurate self-assessment empowers patients to take an active role in their recovery, reinforcing rehabilitation adherence and engagement [5,6,7,9]. The simplicity of patient-reported outcome measures is important in directly communicating a patient’s perceived and actual functional status to their clinician.

The high accuracy of patient self-identification may reflect the cumulative nature of gait perception during daily activities. Unlike brief clinic-based observation, patients experience gait deviations repeatedly in real-world settings, where pain, fatigue, and asymmetry may become more apparent. This lived experience may explain why some patients demonstrated sensitivity to subtle gait changes not readily observed during a short clinical assessment.

The PROMIS Physical Function assessments are a validated tool to assess patients’ functional status, and average t-scores from this tool were shown to differ between limp and no-limp patients by a magnitude greater than minimal clinically important differences noted in prior post-traumatic populations [17,18]. This study estimated a PROMIS-PF score of 37.2 or lower as a cutoff score predictive of limp with a specificity of 89.5% (95 CI: 68.6–97.1%) and PPV of 92.6% (95 CI: 76.6–97.9%). Combining a simple self-report of limp status with PROMIS-PF scores could serve as a low-cost, easily deployable screening tool for use in postoperative follow-up. In telemedicine or resource-limited environments, this combination of assessments could help triage patients who require in-person assessment while safely monitoring those progressing well. Such strategies could reduce unnecessary clinic visits, accelerate intervention for those with functional decline, and ultimately improve outcomes.

As orthopedic care increasingly incorporates telemedicine and remote monitoring, low-burden patient-reported measures may play an essential role in postoperative surveillance [19]. Prior clinical studies have demonstrated that mobile digital health platforms can improve patient engagement and postoperative outcomes; Venkatraman et al. reported improved monitoring among spine surgery patients using a mobile health platform [20], while Milliren et al. showed that higher digital engagement was associated with improved outcomes following total joint arthroplasty [21]. Additional mHealth research has further demonstrated that patient engagement correlates with perceived treatment effectiveness, reinforcing the clinical relevance of simple patient-reported inputs [22,23]. Structured assessment tools have similarly been shown to improve quality in orthopedic practice, underscoring the value of standardized, reproducible measures in routine care.

This study may be the first orthopedic trauma study to establish the accuracy of patient self-perception of a limp. Prior work in the neurological literature has linked self-reported unsteadiness to measurable gait deviations [24]. However, this finding has not been validated in the postoperative orthopedic trauma population, and a correlation between limp and functional status by validated patient-reported outcome measures has not previously been established.

The sample size in this study became modest due to strict inclusion criteria, yet the statistical significance and effect sizes observed suggest that these findings are robust. While these findings are statistically strong, studies with larger and more heterogeneous sample size, and inclusion of individuals who experienced polytrauma could build on this work to strengthen generalizability and external validity. Additionally, multiple blinded raters and used of validated gait metrics would improve the objectivity and generalizability of this analysis. Thus, the results of this study are susceptible to systematic rater bias and inter-rater reliability could not be assessed. Future studies should also expand beyond binary classification to evaluate whether patients can distinguish between subtle and obvious gait abnormalities and whether patient self-recognition of gait disturbance predicts long-term functional outcomes, delayed recovery, or the need for additional intervention. Longitudinal assessment across standardized postoperative time points may clarify how changes in patient-perceived gait correlate with objective recovery milestones and validated functional measures. Further work examining injury-specific patterns of gait recovery and incorporating multiple evaluators or quantitative gait metrics may help refine the role of patient-reported gait assessment as a scalable screening and triage tool in orthopedic trauma care.

## 5. Conclusions

This study demonstrates that patients recovering from lower extremity trauma can accurately recognize the presence or absence of gait disturbances during postoperative recovery. The presence of a limp was associated with lower PROMIS Physical Function scores, indicating worse self-reported functional status. These findings suggest that patient self-awareness of gait abnormality may serve as a clinically meaningful adjunct to postoperative assessment. Incorporating patient self-assessment into routine follow-up may support proactive care, enhance patient engagement, and facilitate timely rehabilitation adjustment.

## Figures and Tables

**Table 1:** Contingency Matrix of Patient and Clinician Agreement on Limp Presence or Absence.

		Patient Self-Identified	
		No Limp	Limp
Clinician-Observed	No Limp	16	3
	Limp	1	46

**Table 2:** Agreement and accuracy metrics for the entire cohort and stratified by follow up time from surgery.

	Total	0–52 Weeks	>52 Weeks
Estimate (95% CI)	(*n* = 66)	(*n* = 38)	(*n* = 28)
Sensitivity	97.9% (88.9–99.6%)	96% (80.5–99.3%)	100% (85.1–100%)
Specificity	84.2% (62.4–94.5%)	100% (77.2–100%)	50% (18.8–81.2%)
PPV	93.9% (83.5–97.9%)	100% (86.2–100%)	88% (70.0–95.8%)
NPV	94.1% (73.0–99.0%)	92.9% (68.5–98.7%)	100% (43.9–100%)
Overall accuracy	93.9% (85.4–97.6%)	97.4% (86.5–99.5%)	89.3% (72.8–96.3%)
Cohen’s kappa	0.84 (0.70–0.99)	0.94 (0.83–1)	0.61 (0.23–0.99)

Agreement metrics between patient-reported and clinician-observed limp status. **PPV:** positive predictive value. **NPV:** negative predictive value. **95% CI:** 95% confidence intervals.

## Data Availability

The original contributions presented in this study are included in the article/Supplementary Material. Further inquiries can be directed to the corresponding author.

## Supplementary Materials

The following supporting information can be downloaded at: https://www.mdpi.com/article/10.3390/jcm15031175/s1, Table S1: Demographic and clinical characteristics of the study cohort, including age, sex, date of surgery, time from injury, injury category, specific injury, comorbidities, pain score, PROMIS-PF t-scores, and presence of limp.

## Abbreviations

The following abbreviations are used in this manuscript:

PROMIS-PF	Patient Reported Outcomes Measurement Information System- Physical Function
PPV	Positive predictive value
NPV	Negative predictive value
95% CI	95% Confidence interval
